# Verification of a Central Pacemaker in Brain Stem by Phase-Coupling Analysis Between HR Interval- and BOLD-Oscillations in the 0.10–0.15 Hz Frequency Band

**DOI:** 10.3389/fnins.2020.00922

**Published:** 2020-08-28

**Authors:** Gert Pfurtscheller, Andreas R. Schwerdtfeger, Beate Rassler, Alexandre Andrade, Gerhard Schwarz, Wolfgang Klimesch

**Affiliations:** ^1^Institute of Neural Engineering, Graz University of Technology, Graz, Austria; ^2^BioTechMed Graz, Graz, Austria; ^3^Institute of Psychology, University of Graz, Graz, Austria; ^4^Carl-Ludwig-Institute of Physiology, University of Leipzig, Leipzig, Germany; ^5^Institute of Biophysics and Biomedical Engineering, Faculty of Sciences of the University of Lisbon, Lisbon, Portugal; ^6^Division of Special Anaesthesiology, Pain and Intensive Care Medicine of Department of Anaesthesiology and Intensive Care Medicine, Medical University of Graz, Graz, Austria; ^7^Centre for Cognitive Neuroscience, University of Salzburg, Salzburg, Austria

**Keywords:** central pacemaker, brain stem, heart rate interval, BOLD oscillations, neurovascular coupling

## Abstract

The origin of slow intrinsic oscillations in resting states of functional magnetic resonance imaging (fMRI) signals is still a matter of debate. The present study aims to test the hypothesis that slow blood oxygenation level-dependent (BOLD) oscillations with frequency components greater than 0.10 Hz result from a central neural pacemaker located in the brain stem. We predict that a central oscillator modulates cardiac beat-to-beat interval (RRI) fluctuations rapidly, with only a short neural lag around 0.3 s. Spontaneous BOLD fluctuations in the brain stem, however, are considerably delayed due to the hemodynamic response time of about ∼2–3 s. In order to test these predictions, we analyzed the time delay between slow RRI oscillations from thorax and BOLD oscillations in the brain stem by calculating the phase locking value (PLV). Our findings show a significant time delay of 2.2 ± 0.2 s between RRI and BOLD signals in 12 out of 23 (50%) participants in axial slices of the pons/brain stem. Adding the neural lag of 0.3 s to the observed lag of 2.2 s we obtain 2.5 s, which is the time between neural activity increase and BOLD increase, termed neuro-BOLD coupling. Note, this time window for neuro-BOLD coupling in awake humans is surprisingly of similar size as in awake head-fixed adult mice (Mateo et al., 2017).

## Introduction

Slow BP oscillations in the frequency range around 0.1 Hz are known as Mayer waves and well documented [see, review by Julien (2006)]. However, it is still unknown how they are generated. Baroreflex mechanisms and intrinsic activity of sinoatrial node cells may play a dominant role (Van Roon et al., 2004; Moen et al., 2019), but also a central (neural) pacemaker may contribute independently to their generation (Preiss and Polosa, 1974; Zhang et al., 1998; Eckberg et al., 2016; Ghali and Ghali, 2020). This has been controversely discussed a couple of years ago (Eckberg and Karemaker, 2009). Support for the central pacemaker hypothesis comes from the work of Perlitz et al. (2004). They observed a neural brain stem rhythm with frequencies around 0.15 Hz (emerging in reticular neurons in lower brain stem) which was phase coupled with respiration and heart rate beat-to-beat interval (RRI) fluctuations in dogs as well as humans (e.g., Lambertz and Langhorst, 1998; Perlitz et al., 2004). The brain stem seems to be a reasonable location for a central pacemaker, because it accommodates the cardiovascular and respiratory centers and is the origin of the ARAS (Moruzzi, 1958), a network arising from the lower brain stem. The ARAS is dynamically organized and modulates distant cortical areas. Several studies indicate that the “0.15-Hz rhythm” of Perlitz et al. (2004) may not be confined to this single frequency, but comprises slow rhythms with frequency components of 0.15 ± 0.03 Hz. Kuusela et al. (2003) analyzed BP and HR signals and observed two distinct frequency components, one around 0.08 Hz and another around 0.12 Hz. Moreover, Rassler et al. (2018) found alternating 0.10-Hz and 0.15-Hz waves in RRI and respiration signals, and Yuen et al. (2019) reported on blood oxygenation level-dependent (BOLD) oscillations that most likely are associated with vasomotor activity centered at 0.15 Hz. Together, these studies document the importance of frequency components >0.1 Hz in the brain.

In the present study, we analyze time delays (using the phase locking value, PLV; Lachaux et al., 1999) between RRI fluctuations and BOLD signals in the frequency range of 0.10–0.15 Hz. Due to the close interaction between brain and heart (see review, Thayer and Lane, 2009), slow changes in neural activity are linked to slow RRI changes. Thus, RRI oscillations can be used to index central nervous system activity. Because neural oscillations cannot be recorded from the brain stem of humans, we instead record BOLD signals from the brain stem and predict the time lag on the basis of the following working hypothesis:

*If slow rhythmic neural activity (central pacemaker) exists in brain stem, then two responses are expected: slow oscillating cardiac RR intervals and slow oscillating BOLD signals in brain stem.* Specified: If a neural pacemaker generates oscillations in a particular frequency band (e.g., 0.10–0.15 Hz), it should modulate slow cardiac interval (RRI) fluctuations rapidly due to fast vagal innervation (within 0.2–0.3 s; Smith, 1974). Moreover, it should affect BOLD fluctuations in the brain location of interest, which are, however, delayed by a few seconds due to the hemodynamic response. This hemodynamic response is often termed neurovascular coupling (Arthurs and Boniface, 2002), however, this term can be misleading. Therefore, it is suggested to use the term neuro-BOLD coupling (NBC), which defines the time between neural activity (e.g., gamma power) increase and BOLD increase. Mateo et al. (2017) reported a NBC time of 2.6 s in mice. This means, the pattern of the cardiac interval time series (RRI signal) and the pattern of the BOLD signal should coincide wave-by-wave with a time-shift of 2–3 s.

If this hypothesis is correct, a method is available to assess sources of neural oscillations in the brain stem indirectly by analyzing the phase coupling between RRI and BOLD signals in the sense of a surrogate indicator.

Taken together, the two main questions guiding this research were: (i) Does a “central pacemaker” exist in the brain stem and contribute to the generation of RRI oscillations? (ii) Have BOLD signals in a frequency range above 0.10 Hz a neural origin?

## Materials and Methods

### Participants

A total of 23 participants (12 female, 22 right-handed) between 19 and 34 years (mean ± SD: 24 ± 3.2 years) took part in the study. All were naïve to the purpose of the study, had no former MRI experience, had normal or corrected-to-normal vision and were without any record of neurological or psychiatric disorders, as assessed by self-report. All participants gave informed written consent to the protocol of the study, which was approved by the local Ethics Committee at the University of Graz (number: GZ. 39/75/63 ex 2013/14).

The experimental task consisted of four resting states (R1, R2, R3, R4) and four within-scanner questionnaires (AS1, AS2, AS3, AS4), carried out in two sessions separated by about 50 min. The first session (a) started with the first questionnaire (AS1) and was followed by the first resting state (R1). Thereafter, two motor tasks, unrelated to this study, were performed. The first session ended with the second resting state (R2) and second questionnaire (AS2). Filling out each questionnaire took approximately 5 min and each resting state lasted for about 350 s. The second session (b) was quasi a duplicate of the first one, with two resting states (R3, R4) and two within-scanner questionnaires (AS3, AS4). Individuals were requested to keep their eyes open, stay awake, and avoid movements during the resting states.

State anxiety was assessed with the state-trait anxiety and depression inventory (STADI; Laux et al., 2013), which was presented on a screen within the scanner. The STADI is an instrument constructed to assess both state and trait aspects of anxiety and depression. It is based on the State-Trait Anxiety Inventory (Spielberger et al., 2009), but allows a reasonable separation of anxiety and depression symptoms. Items were answered with a trackball following each resting state.

### Recording of Physiological Signals (ECG, Respiration) and RRI Time Courses

Electrocardiogram and respiration were recorded inside the scanner using the PMU (Siemens Physiological Measurement Unit). For the positioning of the ECG electrodes on the thorax, standard channels (Siemens Standard, lead 1) were used. The respiratory data were acquired using a pneumatic cushion connected via an air hose to a pressure sensor on the recording unit. The cushion was attached to the subject by using a respiration belt. This technique allowed a wave-by-wave determination of respiratory rate. Sampling rate was 400 Hz. QRS detection and subsequent computation of RRI time series was performed using fMRI plug-in for EEGLAB (Niazy et al., 2005). Each interval was referred to the previous R-wave (instantaneous interval; De Boer et al., 1985). In order to assure a reasonable quality of the ECG recording during a fMRI high scanning rate, RRI signals were re-processed using the Kubios HRV Premium Package (Kubios Ltd., Finland; version 3.0.2; Tarvainen et al., 2014). The RRI time series were subsequently resampled to the same sampling frequency of the BOLD time series. For the display and identification of local peaks in BOLD and RRI signals a resampling rate of 10 Hz was used.

### Resting State fMRI and BOLD Signals

Resting-state functional images were acquired with a 3T scanner (Magneton Skyra, Siemens, Erlangen, Germany) using a multiband GE-EPI sequence (Moeller et al., 2010) with a simultaneous six-band acquisition with TE/TR = 34/871 ms, 52° flip angle, 2 × 2 × 2 mmł voxel size, 66 contiguous axial slices (11 × 6), acquisition matrix of 90 × 104 and a FOV of 180 × 208 mm^2^, 400 volumes.

Pre-processing resorted to the DPARSF toolbox (Yan and Zang, 2010) and included removal of the first 10 volumes, slice timing correction adapted for multiband acquisitions (Woletz et al., 2014), rigid-body motion correction, normalization to Montreal Neurological Institute (MNI) space, resampling to 2-mm isotropic voxels, spatial smoothing with a 4-mm FWHM Gaussian kernel, linear detrending and extraction of time series from various regions of interest (ROIs) using the AAL atlas (Tzourio-Mazoyer et al., 2002).

### Averaging of Breathing, RRI and BOLD Waves in Resting States

**FIGURE 1:**
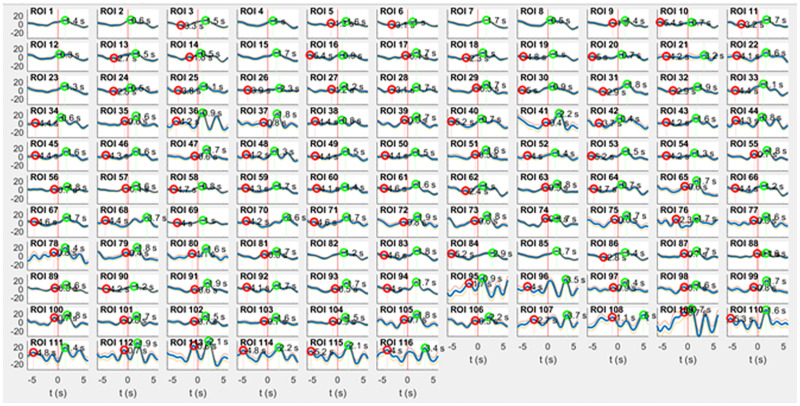
RRI peak-triggered slow BOLD waves (mean ± SE) from 116 ROIs during rest. Respiratory artifacts of comparably large amplitude are prevalent in ROI 36 (right cingulum, supplied by anterior cerebral artery), ROIs 95, 96 (pons/brain stem, supplied by basilar artery) and ROIs 109–116 (vermis cerebelli, also supplied by basilar artery). The majority of ROIs exhibits artifact-free signals. Surprisingly, even a few ROIs in pons/brain stem (e.g., ROI 91 and 93) are of satisfying signal quality.

Averaging can be used to enhance the signal-to-noise ratio of physiological signals but it requires a trigger. Because stimuli-based triggers are not available in resting state data, the periodic positive peaks (maximal beat-to-beat intervals) of the RRI signal were used to define triggers (Pfurtscheller et al., 2017, 2019). This procedure includes the following steps: First, inspection of the RRI time course with slow spontaneous fluctuations and marking of a few positive peaks preferably occurring in intervals larger than 6 s and shorter than 12 s. Second, using these peaks as trigger to calculate averages (mean ± SE) of breathing, RRI and BOLD waves over a time frame between 6 s before and 6 s after the trigger (examples see Figures 1, 4).

### Search for Artifact-Free ROIs

For the anatomical leveling, the AAL atlas (Tzourio-Mazoyer et al., 2002) was used, and 116 ROIs were defined. The main benefit of the ROI over the voxels-level approach is the increased signal-to-noise ratio. Each ROI signal is computed by averaging the BOLD signal over hundreds of voxels. ROIs 1–90 correspond to cortical and subcortical structures, ROIs 91 – 108 to regions in the cerebellum including brain stem, and supplementary ROIs 109–116 to the vermis cerebelli. Due to the proximity of cerebellum and brain stem in axial slice and due to the contiguity of a major artery, it is reasonable to assume that the signals collected from AAL ROIs labeled “cerebellum” include the upper brain stem with the pons. Therefore, in this paper the denotation pons/brain stem is used for recordings from ROIs 91–108 instead of “cerebellum.”

ROIs with even numbers correspond to the right, and those with odd numbers to the left hemisphere. A major challenge is the vascular pulsing related to the main cerebral arteries, especially in the pons/brain stem. Therefore, a search for respiratory artifacts in all 116 ROIs was conducted through calculating averaged BOLD waves based on the slow waves in RRI signals. Images of 116 averaged BOLD waves (mean ± SE) from cortex, subcortex and pons/brain stem from one representative subject (9R1) with a spontaneous respiratory rate of ∼ 22 breaths/minute are shown in Figure 1. This Figure demonstrates that ROIs without respiratory artifacts are present in a few ROIs in pons/brain stem (e.g., ROIs 91 and 93) similar to ROIs in cortical areas (e.g., precentral gyrus, ROI 1) although the basilar artery is tight-fitting in the pons.

**FIGURE 2:**
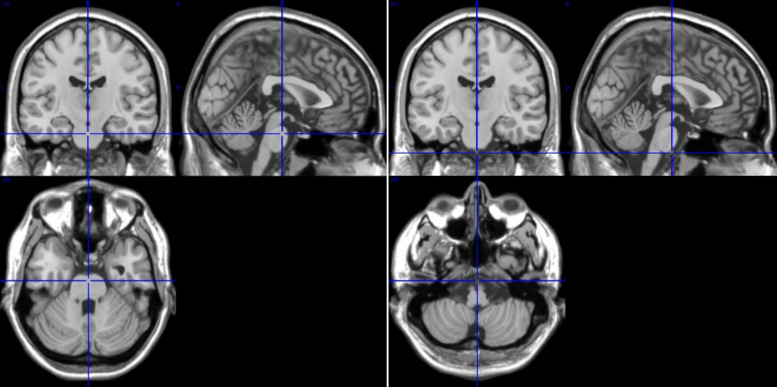
T1-images from medial, sagittal and axial slices from rostral pons ROIs 91/92 (**left**, Talairach space 0, 16, −28) and caudal pons ROIs 107/108 (**right**, Talairach space 0, 16, −44). The difference between ROIs 91/92 and 107/108 is 16 mm.

Based on this type of artifact detection, ROIs 95 and 96 were discarded, and ROIs 107 and 108 were excluded due to the small number of voxels (<200). From the ROIs representing cerebellum and brain stem (ROIs 91–108) 14 ROIs from pons/brain stem were considered for further analyses. These ROIs represent a distance of 16 mm between rostral (ROIs 91, 92) and caudal (ROIs 107,108) parts of pontine structure in upper brain stem (see sagittal and axial slices, Figure 2).

### Calculation of Phase-Locking Between Oscillating RRI and BOLD Signals

Wavelet transform coherence was applied to the BOLD and RRI time series using the “Cross-wavelet and Wavelet Coherence” toolbox (Grinsted et al., 2004). We focus on the phase component, which allows to compute the PLV throughout the acquisition interval, except for small sections at the beginning and end where results are known to be unreliable (Torrence and Compo, 1998). PLV is a normalized measure of how much the phase difference between two signals changes in a user-chosen time window, regardless of the actual phase difference value; the reader is referred to Lachaux et al. (1999) and Pfurtscheller et al. (2017) for more details. The PLV was calculated in the band 0.10–0.15 Hz between RRI time course and the BOLD time series from the remaining 14 ROIs and ROI 1 (left precentral gyrus) for control. Two PLV-based parameters were computed: time delay (TD), which is the phase delay converted to time, and significant length of phase coupling (sigbin), which is the percentage of time samples within the time series that survive a 0.05 significance threshold (i.e., the null hypothesis of no phase-locking rejected with 95% certainty; Pfurtscheller et al., 2017). A positive time delay (pTD) indicates a lead and a negative delay (nTD) a lag of RRI oscillations compared to BOLD oscillations in the brain stem.

Neural processes (e.g., activity of a central pacemaker) are accompanied by changes in blood oxygenation and cerebral blood flow, both detectable through BOLD contrast measurements (Obrig et al., 2000; Buxton et al., 2004). BOLD is a complex function of cerebral metabolism, cerebral blood flow and cerebral blood velocity and sensitive to different types of artifacts as, for example, respiratory motion (Murphy et al., 2013).

The BOLD signals from pons/brainstem are expected to reflect the contribution of local vascular network and one major blood vessel, the basilar artery, which is close to the medulla and the pons. Similarly, important nuclei involved in cardiac control such as, for example, solitary tract nucleus and vagal motor neurons in the nucleus ambiguus and dorsal vagal motor nucleus are localized in the medulla and the pons (Verberne and Owens, 1998; Thayer and Lane, 2009). This means that two important sources for rhythmic BOLD signals are concentrated in the pons/brain stem: cerebral blood flow and neural activity changes.

### Statistical Analysis

*T*-tests for independent measures were conducted in order to compare pTDs and nTDs between recording sites. Furthermore, the distribution of TDs was analyzed using Kolmogorov-Smirnov tests and Chi^2^ tests. Finally, we compared the highest (HA) and lowest state anxiety (LA) across resting states for each individual using independent *t*-tests. Of note, TDs for each individual anxiety score were treated as independent samples. Strictly, these measures constitute dependent measures for each individual, however, aggregating reliable TDs (%sigbin > 10) across locations within individuals would result in a large number of missing cases, which severely attenuates statistical power. It should be noted though that our data analytic approach inflates degrees of freedom, thus constituting a rather liberal statistical approach. We deem this acceptable for a rather exploratory study design, but are aware that the findings warrant replication with larger sample sizes, thus allowing more conservative statistical approaches.

## Results

### Distribution of Time Delays Between Slow RRI and BOLD Oscillations in the Brain Stem

**FIGURE 3:**
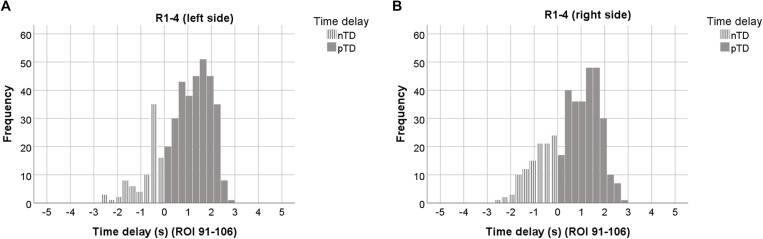
Histograms of TDs averaged over all 4 resting states, plotted separately for the left **(A)**, and right brain side **(B)**. Data from 7 ROIs and sigbin ≥10% are accumulated. ROIs with odd numbers represent the left, ROIs with even numbers the right brain side. Histograms representing pTDs (between 0.1 and 3 s) are indicated by dark gray color.

A time delay (TD) was considered reliable, when the associated significant length of phase coupling (sigbin) was equal to, or larger than 10% (percentage of samples within the time series; arbitrary threshold). TD values and sigbins from all subjects were summarized in two matrices with 23 rows (subjects) and 14 columns (ROIs) for each resting state. For each individual resting state and all states together, histograms of TDs were plotted, and differences were calculated between both brain sides (Table 1). Significant side differences were found for the first resting state (*p* = 0.039) and all four resting states together (*p* = 0.002) with more pTDs in the left side. The AS showed the highest level in the first resting state (R1) with AS = 20 ± 5 and a decline thereafter (Table 1). Interestingly, this high AS in R1 coincides with the left-sided predominance of pTDs in R1. The histograms across all resting states are presented in Figures 3A,B and show a clear dominance of pTDs.

**TABLE 1:** Resting state (R), state anxiety (AS) and time delays in (s) for the left and right brain side for each resting state and all states together (1–4).

			**Sides**						
			**Left**			**Right**			
	***AS***		***Time delay***			***Time delay***			
***R***	***M***	***SD***	***M***	***SD***	***N***	***M***	***SD***	***N***	***p***
1	19.88	4.57	0.91	1.06	121	0.62	1.07	116	0.039
2	16.80	4.42	0.76	1.15	108	0.58	1.09	103	0.244
3	14.68	4.06	0.69	1.12	108	0.49	1.21	96	0.220
4	14.04	4.02	1.05	0.86	87	0.80	0.94	81	0.070
1–4	16.35	3.28	0.84	1.07	424	0.61	1.09	396	0.002

*TDs were determined from ROIs 91–106 with the exception of ROIs 95/96. Data are given as means (M, i.e., averages of individual samples) and SD. N marks the number of TDs, and p denotes the probability of significant differences of TD values between left and right side.*

The histograms in Figures 3A,B are based on ∼ 800 TDs from 23 subjects, 4 resting states and 14 ROIs. They are clearly dominated by pTDs with largest accumulation (peaks) close to +2 s in both sides of the cerebellum/brain stem. The distributions of nTDs and pTDs were significantly different for both the left (Kolmogorov–Smirnov *Z* = 8.18, *p* < 0.001) and right side (Kolmogorov–Smirnov *Z* = 8.83, *p* < 0.001). Furthermore, a Chi^2^ test revealed that the numbers of pTDs and nTDs differed by side (Chi^2^ = 5.65, *p* = 0.02), suggesting that pTDs in the left side were overrepresented and nTDs underrepresented. Most importantly, our findings – as depicted in Figure 3 – show that none of the observed TDs was larger than +/−3 s. Remarkable is not only the predominance of pTDs on the left side but also the general dominance of pTDs in the pons/brain stem.

In summarizing, the analysis of TD histograms revealed the following findings: (i) No TDs were found larger than +/−3 s. (ii) A clear predominance of pTDs was found in the left pons/brain stem. (iii) Significant phase-coupling between RRI and BOLD oscillations was largest and most dominant across subjects in the first resting state and declined thereafter.

### The Search for pTDs in the Brain Stem With Maximal Delays Between RRI and BOLD Oscillations

The accumulation of pTDs close to 2 s in the left pons/brain stem (Figure 3A) and its restriction to less than 3 s is a novel observation and raises questions not only about the incidence of pTDs across subjects, but also about the maximal possible time delay. In order to search for such a SSMTD, a threshold has to be defined. Based on the histogram across all resting states from the left side (Figure 3A) with a sharp drop of the columns to the right of the peak close to 2 s (dark gray), an arbitrary threshold of TD = 1.9 s was defined. For the search process the following three conditions had to be fulfilled: TD ≥ 1.9 s, sigbin ≥20% and at least two positive findings within the 14 ROIs.

The results of the search process in the four resting states are summarized in Table 2. Seven subjects complying with the above conditions were found in R1, three subjects in R2, two subjects in R3 and three subjects in R4. Altogether, 12 different subjects were identified with significant phase-coupling between RRI and BOLD oscillations in the pons/brain stem and a SSMTD = +2.2 ± 0.2 s. These findings show a clear predominance for R1. They primarily comprise the ROIs 93, 99 and 103 and depict slow RRI oscillations leading those in BOLD signals by about 2.2 s. Remarkably, this SSMTD was very consistent across these subjects despite of widely different breathing periods. In these selected subjects, the sigbin values varied between 23 and 82%. With the exception of two subjects (14R4, 3R2) all ROIs with dominant pTDs were left-sided. Noteworthy, the small inter-subject variability of the RRI vs. BOLD time delay (pTD = 2.2 ± 0.2 s) indicates an interesting “landmark.”

**TABLE 2:** Age (years), state anxiety (AS), breath duration (Tresp [seconds], given as means ± SD), time delay (TD [seconds]) and significant phase coupling (%sig) for ROIs with a subject-specific maximal pTD (SSMTD) in pons/brain stem, ROI 1 (precentral gyrus) and ROI 93 in pons/brain stem.

**Subject/state**	**Characteristics**			**SSMTD**			**Precentral gyrus**			**Pons**		
	**Age (*y*)**	**AS**	**Tresp (*s*)**	**ROI**	**TD (*s*)**	**%sig**	**ROI**	**TD (*s*)**	**sigbin**	**ROI**	**TD (*s*)**	**%sig**
1R3	19	16	6.3 ± 3.0	99	2.4	23	1	0.2	27	93	1.4	21
7R1	23	17	5.0 ± 0.8	99	2.3	33	1	1.8	25	93	1.3	33
9R1	27	23	3.1 ± 0.6	93	1.9	36	1	1.4	48	93	1.9	36
11R1	25	25	7.3 ± 2.0	103	2.5	82	1	2.4	54	93	2.3	70
13R2	23	22	2.8 ± 0.3	103	2.0	34	1	2.1	29	93	1.3	46
17R1	24	13	7.2 ± 1.9	93	2.3	47	1	2.4	50	93	2.3	47
18R1	28	28	6.2 ± 2.6	103	2.2	36	1	2.0	54	93	1.9	38
20R1	25	22	3.8 ± 0.7	93	2.3	47	1	1.3	54	93	2.3	47
10R1	24	18	3.1 ± 0.3	97	2.1	40	1	1.6	17	93	1.6	10
3R2	23	16	3.9 ± 0.6	92	2.1	30	1	0.8	23	93	1.8	30
14R4	23	20	3.2 ± 0.6	104	2.1	27	1	1.1	27	93	1.4	27
25R4	20	17	3.5 ± 0.6	93	1.9	56	1	0.5	16	93	1.9	56
**mean**	23.67	19.75	4.59		2.17	40.92		1.47	35.33		1.78	38.42
**SD**	2.43	4.17	1.70		0.18	15.23		0.69	14.63		0.37	15.44

*Data from 12 subjects (first column on the left side).*

For a comparison, pTD calculation was also performed for ROI 93, a region in the pons/brain stem with large sigbins, and ROI 1, representing the left precentral gyrus (Table 2). In both cases, the mean pTD was smaller and its inter-subject variability (SD) higher as compared to SSMTD.

### Slow RRI Oscillations Lead BOLD Oscillations

**FIGURE 4:**
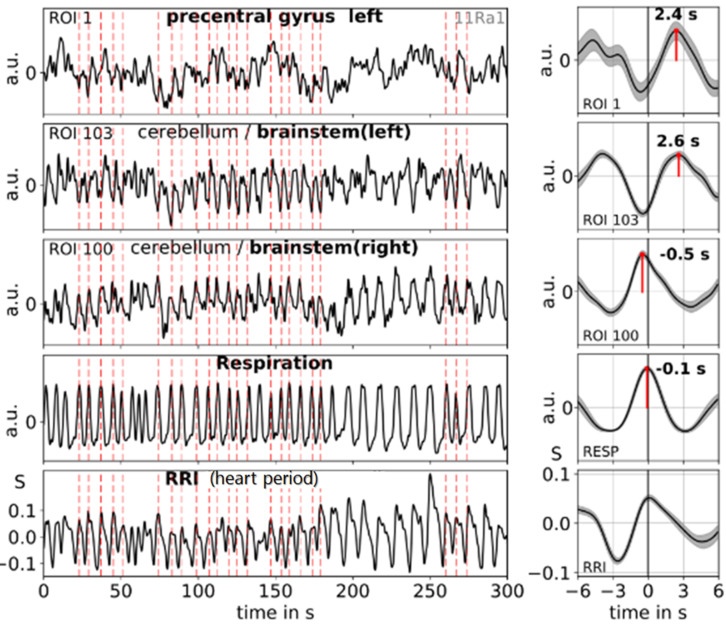
Examples of ongoing BOLD, RRI and respiration signals (1:1 coupling) and corresponding averaged waves (±SE) for one subject (11R1) with dominant 0.10-Hz and 0.15-Hz oscillations. The vertical dashed lines indicate maxima (peaks) of waves with a period of ∼7 s of RRI oscillations used as trigger for averaging. Peaks of the averaged waves are indicated. Note, the peak difference between RRI and BOLD (ROI 103) waves of about 2.6 s, the coincidence of RRI, respiration and BOLD (ROI 100) waves and the cessation of respiratory sinus arrhythmia (nonRSA). Modified from Pfurtscheller et al. (2019).

RRI and BOLD signals have quite different origins. One is recorded from the thorax (RRI) and composed of successive cardiac beat-to-beat intervals measured in seconds. The other signal (BOLD) is recorded from the pons/brain stem and is composed of different neural and non-neural components (Obrig et al., 2000; Buxton et al., 2004; Murphy et al., 2013). It may be helpful to illustrate this novel approach by depicting the signals (also including respiration) in one characteristic subject (11R1), thus highlighting the dynamic and coincidence of various physiological signals (Figure 4). Subject 11R1 exhibits a 1:1 coupling between RRI and respiration signals. The graph shows a dominant coincidence of slow waves in RRI, respiration and BOLD signals from ROI 100 (right side). Furthermore, a delay of 2–3 s in BOLD waves from pons/brain stem (ROI 103, left side) and precentral gyrus (ROI 1) in comparison to RRI waves is shown. This delay of averaged BOLD waves (Figure 4, right panel) corresponds quite well with the pTD values in Table 2 (ROI 103: pTD = 2.5 s; ROI1: pTD = 2.4 s). The sigbin values (82 and 54%) underline the relative long-lasting stability of the phase coupling in this subject, although the dominant period of breathing waves (about 80% of all recorded breaths) varied between 7 and 10 s.

The examples shown in Figure 4 demonstrate that two types of spontaneous slow BOLD oscillations in the pons/brain stem can be observed, one of presumably neural origin and another of non-neural origin. The former is delayed (relative to the RRI signal) by 2–3 s in the case of 1:1 coupling (one breath per RRI cycle), the latter is in-phase with respiration (see also, Pfurtscheller et al., 2019). In subjects with a normal resting respiration rate (e.g., of 0.3 Hz and about three breaths per RRI cycle of 9 s), a similar pattern was found with a delay of 2–3 s between slow RRI and BOLD oscillations in pons/brain stem and precentral gyrus.

### pTDs and State Anxiety

**FIGURE 5:**
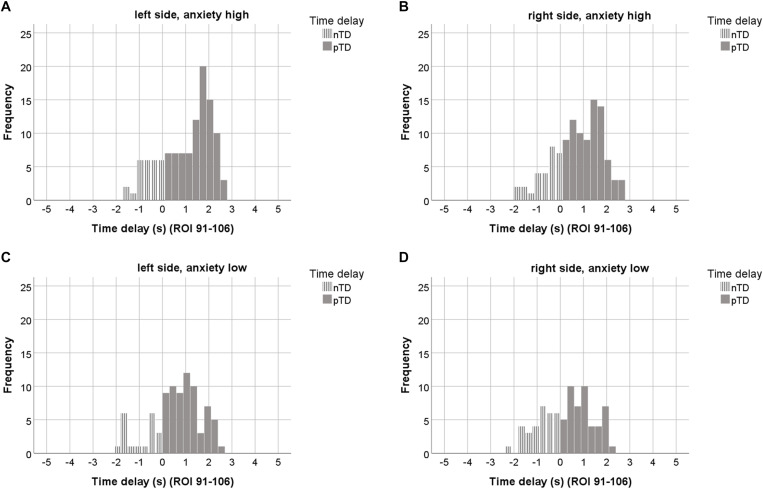
The upper row depicts histograms of TDs from the “high anxiety” category (23 subjects, 7 ROIs out of ROIs 91–106) for the left **(A)** and right side **(B)**. The lower row shows TD histograms from the “low anxiety” category for left **(C)** and right side **(D)**. ROIs with odd numbers represent the left, ROIs with even numbers the right brain side. Histograms representing pTDs (between 0.1 and 3 s) are indicated by dark gray color.

An interesting question is the functional meaning of increased pacemaker activity. To clarify this, the AS of the 23 subjects was investigated in relation to the pTD values dominating in both sides. Across resting states, the highest and lowest anxiety score was identified for each subject, thus resulting in two anxiety categories (high anxiety and low anxiety). In most cases, the highest AS score was found in the first resting state (Chapman et al., 2010; Pfurtscheller et al., 2018). However, there were 6 subjects with elevated AS in one of the subsequent resting states. The pTDs from both anxiety categories are summarized in Table 3 and visualized as histograms in Figure 5. Anxiety categories significantly differed in AS (*p* < 0.001), and there was a significant difference between the pTD values on the left side (*p* < 0.001). High anxiety was accompanied by a higher pTD as compared to low anxiety. The difference of pTDs between left and right brain side was also significant for high anxiety only (*p* = 0.006), suggesting a left side dominance. Additional analyses were conducted to compare differences in the distribution of TDs between high and low anxiety categories using Kolmogorov-Smirnov tests for two samples. Results revealed that pTDs from low and high anxiety category, respectively, seem to stem from different populations (*Z* = 2.40, *p* < 0.001). These findings may suggest that a pacemaker activity (large pTDs) may be associated with the processing of anxiety.

**TABLE 3:** Anxiety category (AC), state anxiety (AS), positive time delays (pTD) in (s) for the left and right brain side for “high anxiety” (HA) and “low anxiety” (LA).

			**Sides**						
			**Left**			**Right**			
**AC**	***AS***		***p Time Delay***			***p Time Delay***			
	***M***	***SD***	***M***	***SD***	***N***	***M***	***SD***	***N***	***p***
HA	20.87	4.83	1.53	0.65	88	1.25	0.65	81	0.006
LA	13.48	3.07	1.10	0.65	66	1.03	0.58	48	0.552
Diff	7.39		0.43			0.23			
*p*	<0.001		<0.001			0.051			

*pTDs were determined from ROIs 91–106 with the exception of ROIs 95/96. Data are given as means (M) and SD, N marks the number of pTDs. The *p*-values in the right column indicate differences in pTD values between left and right side; *p*-values in the bottom row indicate differences between HA and LA categories.*

## Discussion

Phase locking analysis between RRI and BOLD signals in the pons/brain stem revealed several interesting findings that support our pacemaker hypothesis. The findings that the time windows of the time delays (TDs) between RRI and BOLD never exceeded 3 s and that their maximal time delay (“landmark”) showed a quite precise mean ± SD of pTD = 2.2 s ± 0.2 s in 50% of the sample with a left-sided dominance in the first resting state are particularly noteworthy.

### BOLD Signals in Brain Stem

Our interest is directed to the upper brain stem with the pons and the basilar artery as one of the major brain arteries directly ahead. The pons has a rostro-caudal length of ∼2.5 cm and a diameter of ∼ 3 cm in humans (Koehler et al., 1985), includes specific nuclei (e.g., the parabrachial nuclei and locus coeruleus, two structures pivotal for control of cardiovascular activity; Davern, 2014). Furthermore, nuclei of the reticular formation network originating in the lower brain stem seem particularly important. The AAL atlas (Tzourio-Mazoyer et al., 2002) allows to study BOLD signals in 14 ROIs (7 on each side) composed of many voxels in the upper brain stem with a spacing of 2 mm, of which ROIs 91and 92 represent the rostral part and ROIs 107 and 108 the caudal part of the pontine structure (see Figure 2). Studying the specific nuclei in pontine structure needs disentangling signals from noise carefully and cleaning-up of BOLD signals (Brooks et al., 2013).

Unspecific reticular neurons might be involved in the generation of slow rhythms in the pons/brain stem. Lambertz and Langhorst (1998) studied hundreds of reticular neurons in the lower brain stem (medulla oblongata) of adult dogs and observed spontaneous discharges at almost 0.15 Hz in more than 50%. Reticular neurons have also been found in the upper brain stem (pons). Projections from pontine reticular formation to the thalamus have been localized by diffuse tensor imaging (Yeo et al., 2013). All these reticular neurons are part of a diffuse ascending network in lower and upper brain stem and play an important role not only in maintaining behavioral arousal and consciousness (Moruzzi, 1958) but also in control of cardiovascular activity. Pontine structures are also activated during slow breathing (Critchley et al., 2015) and it is concluded that pontine neural activity is essential for physiological coupling of centrally generated respiratory and cardiovascular activities (Eckberg and Karemaker, 2009). The presence of Mayer waves in brain stem sympathetic-related and non-sympathetic-related cells was discussed by Ghali and Ghali (2020).

Prerequisites for the verification of a slow rhythmic neural source in the pons/brain stem are the availability of an associated slow hemodynamic oscillation (neural BOLD signal) delayed by the NBC and the existence of a type of reference signal. Such a reference signal can be either (in rare occasions such as during slow breathing) a non-neural BOLD signal time-locked to slow rhythmic motion of the basilar artery (vascular artifact) or the time-course of cardiac beat-to-beat intervals (RRI) linked to the neural activation and associated with vagal function. Examples of respiratory artifacts are accentuated in Figure 1 close to the cingulum (ROI 36) and in the pons/brain stem (ROIs 95 and 96). The cingulum is surrounded by large blood vessels of the anterior cerebral artery and shows high perfusion (Birn et al., 2006). An almost identical artifact is generated by the basilar artery in the pons/brain stem. A BOLD signal can be used as reference, however, only in the case of spontaneous slow breathing and cessation of RSA (also termed as “switch-off” of RSA, nonRSA; Pfurtscheller et al., 2019). Figure 4 depicts an example with coherent patterns of breathing and BOLD signals in the pons/brain stem (ROI 100). This respiratory artifact in the pons/brain stem can be used to monitor the cyclic start of vasomotion of the basilar artery. Notably, at the same moment of time a delayed neural BOLD signal can be observed in a short distance (e.g., ROI 103 in Figure 4). This example in Figure 4 highlights the existence of two different types of slow BOLD signals in close proximity in the upper brain stem in the case of slow spontaneous internally controlled breathing.

When rhythmic neural activation and control of cardiac function are closely linked, hemodynamic oscillations provoked by rhythmic neural activity changes are also coupled with fluctuations of the cardiac beat-to-beat intervals. Measuring of phase-coupling between RRI and BOLD signals in the 0.10–0.15 Hz band is independent of the respiratory rate and is not based on the selection of epochs with slow RRI fluctuations. Therefore, it can be applied to all BOLD data. Nevertheless, it is remarkable that such similar pTDs with a small variance (pTD = 2.2 s ± 0.2 s; *N* = 12) were observed in different pons/brain stem ROIs separated by few millimeters (see Table 2). Note, data of 5 subjects (part of our cohort) revealed a time delay between two BOLD signals in pons/brain stem of 2.6 ± 0.4 s (Pfurtscheller et al., 2019), meaning that two completely different approaches revealed the about same result.

The availability of various BOLD signals in a close proximity of a few millimeters in pons/brain stem suggests that the neural oscillatory source is not focused and not stable in a specific localization but has its origin very likely in a dispersive neural network (Ramirez et al., 2004) in the brain stem. Neural network dynamics are not fixed but depend on the state of the network or on the action of neuromodulators (Peña-Ortega, 2012), which is in accordance with our observations. The SSMTD of 2.2 ± 0.2 s documents that the time delay between RRI and almost undisturbed neural BOLD oscillations in the pons/brain stem is relatively rare. In the majority of cases, there exists a superposition of neural and non-neural waves, resulting in histogram peaks of pTDs between 1 and 2 s (Figure 3). This may be, at least in part, an explanation for the great inconsistency of time delays between RRI and BOLD signals.

Although a maximal pTD was documented in pons/brain stem, a time delay of similar size was observed in distant ROIs as for example in precentral gyrus. This is not unexpected considering that the ARAS is localized in the brain stem. The observation of similar intrinsic slow waves in ROIs representing pons/brain stem and precentral gyrus suggests a similar neural source. However, further studies are necessary to search for the origin of this neural source. Activation of cortical areas cannot completely be excluded during fMRI-induced anxiety and associated emotional breathing. Behavioral influences during uncomfortable supine position with the head in a limited noisy space may activate primary sensory areas in cortex prior to respiratory neurons in brain stem (Homma and Masaoka, 2008).

### Hemodynamic Delay and pTD

It is important to distinguish between the hemodynamic response function (HRF) used for BOLD corrections in fMRI connectivity studies and the hemodynamic delay characteristic for NBC during rest. In the former case the participant is requested to focus attention to a stimulus of different modality. Therefore, the stimulus-evoked response is affected by stimulus processing, depends on stimulus modality and peaks at 4 – 5 s (West et al., 2019). In contrast, NBC indicates the relationship between slow spontaneous neural and hemodynamic (BOLD) oscillations. Hence, it could be expected that the NBC is shorter than HRF. Interestingly, short delays in humans were found by Fox et al. (2007) and, moreover, in mice (Mateo et al., 2017). Fox et al. (2007) found spontaneous BOLD signals of significantly different magnitude 2 to 3 s after soft vs. hard button pressing in humans. In an experimental trial with awake head-fixed adult mice (Mateo et al., 2017), the entrainment of arteriole vasomotor fluctuations at ∼ 0.1 Hz by neural activity was accompanied by BOLD oscillations about 2.6 s later.

The reported time delay of pTD = 2.2 ± 0.2 s indicates that RRI oscillations precede those in BOLD signals by 2–3 s or, in other words, the longest RRI is followed by a BOLD maximum about 2–3 s later. Inasmuch stimulation of the vagus nerve requires 0.2–0.3 s to produce HR slowing (Smith, 1974), a hemodynamic delay between neural and BOLD oscillations of almost 3 s is therefore realistic.

Although mice and humans differ considerably in body size, vasomotion (a natural collective oscillation of contractile tone in smooth muscles of arterioles) and neural fluctuations (beta/alpha power or envelope over gamma power) seem to be similar and occur at frequencies around 0.1 Hz both in mice (Drew et al., 2011; Mateo et al., 2017) and humans (Obrig et al., 2000; Pfurtscheller et al., 2012). Therefore, it is reasonable to assume that hemodynamic delay is similar, too.

For the investigation of neural-BOLD coupling, the in-vivo animal models have been widely used and essentially increased physiological knowledge (Shtoyerman et al., 2000). The background is that the topological matrices of specific animal brain networks are comparable with those of humans as shown by studies with various designs in rats (Liang et al., 2011; Ma et al., 2018), mice (Scholtens et al., 2018) and Macaque monkeys (Vincent et al., 2007). One of the pioneers in the study of neurovascular coupling by recording of ECoG and regional cerebral blood flow in anesthetized rats was Golanov et al. (1994). The time delay between the onsets of an ECoG burst and slow cerebrovascular wave varied between 1 and 3 s.

### Time Delays Between Slow RRI and BOLD Oscillations Never Exceed ±3 s

The calculation of phase-coupling between physiological signals (RR-interval, pulse-interval, respiration) and fMRI signals (either voxel-based or ROI-based) is an established method (Lachaux et al., 2012; Pfurtscheller et al., 2017; Shokri-Kojori et al., 2018). Investigations of time delays in the 0.07–0.13 Hz band between RRI signals and ROIs in cortical and subcortical areas revealed delays between +3 s and −3 s (Pfurtscheller et al., 2017). Phase lag studies for low frequency fluctuations (<0.1 Hz) between pulse interval and respiratory signals, and voxels in cortical and subcortical areas exhibited mean phase lags between +1.21 ± 1.71 rad (mean ± SD) and −1.55 ± 1.07 rad (this corresponds to +1.9 s and −2.7 s for frequencies of 0.1 Hz) in 203 participants. These studies show that time delays between fMRI and physiological signals include a limited range of a few seconds.

Histograms of TDs are an appropriate way to visualize different features as for example the occurrence of dominant oscillations and specific limits. The histograms in Figure 3 are a catchy document of the prevalence of positive TDs with a peak between 1 and 2s and of the clear limitation within the range of ±3 s in pons/brain stem. This is notable because histograms of TDs from cortical and subcortical ROIs represent two clear peaks, one close to + 2s and another close to – 1s (not shown). While two peaks indicate the existence two dominant BOLD oscillations of neural and vascular origin, one peak of positive TDs refers to a dominance of neural BOLD oscillations interconnected with intrinsic rhythmic neural activity.

**FIGURE 6:**
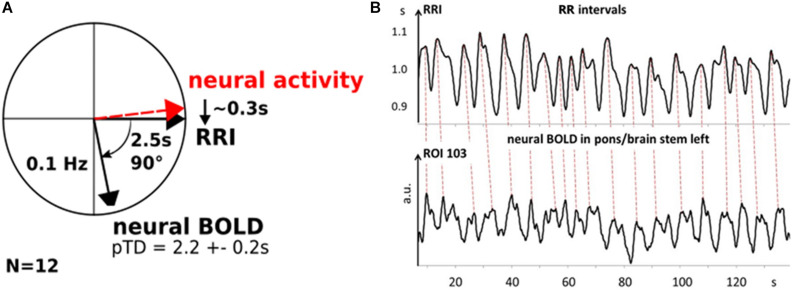
Phasor representation of neural, RRI and BOLD oscillations from 12 subjects with a time delay of 2.2 ± 0.2 s between RRI and BOLD oscillations **(A)**. Neural oscillatory activity leads RRI oscillations by ∼0.3 s. **B**: Patterns of RRI recorded from thorax and BOLD oscillations in pons/brain stem from subject 11R1. Note, RRI oscillations recorded on thorax precede those in BOLD signals in left pons/brain stem (ROI 103) by ∼2.2 s (dashed lines).

In order to visualize phase relationships between slow BOLD, RRI and neural oscillations, the phasor representation (Figure 6A) may be helpful (Zheng et al., 2010; Pfurtscheller et al., 2017). One cycle of 360° represents one 10-s period of a 0.1-Hz oscillation, and 90° phase-shift corresponds to TD of 2.5 s. Figure 6A shows the phasor representation of the relationship between neural, RRI and BOLD oscillations in 12 subjects, and Figure 6B depicts patterns of RRI signals from thorax and BOLD oscillations from pons/brain stem during rest. The strong coincidence between both oscillatory patterns with the delayed BOLD oscillations by 2–3 s points to a neural pacemaker in brain stem.

### Preponderance of pTDs in Left Pons/Brain Stem

The comparison of TD histograms across all resting states and subjects (Figure 3) revealed a significant side-difference (*p* < 0.001) with a dominance of pTDs on the left side. This asymmetry is largest in the first resting state and in accordance with the highest anxiety level (Table 1). The left-sided predominance of pTDs appears amplified in the “high anxiety” category and is accompanied by an elevated HR variability (HRV), and hence might provide resources for the processing of anxiety (Thayer and Lane, 2009). In contrast, a dominant activity of the sinoatrial node is associated with a reduced HRV and a diminished response to external autonomous stimulation (Moen et al., 2019).

The dominance of neural BOLD oscillations >0.1 Hz in the left side of the pons/brain stem is particularly noteworthy. Note, all subjects, except two, were right-handed. The vagus nerve plays an important role in fast cardiac control, and the parasympathetic (vagal) cardiac activity seems to be controlled by the left hemisphere (Craig, 2005). Amygdala studies also confirm the left hemisphere dominance (Baas et al., 2004).

### Why Did Only 50% of the Subjects Show a Maximal pTD of 2.2 ± 0.2 s?

This question warrants some further thoughts. Note, Table 3 and Figure 5 provide evidence that a pacemaker activity (large pTDs) may be associated with the processing of anxiety during unpleasant settings, such as fMRI scanning. However, individuals may adopt different behavioral strategies to cope with such situations. For example, a breathing rhythm around 0.10 Hz has been reported to increase HRV and to facilitate emotional well-being (Lehrer, 2013; Mather and Thayer, 2018). Such a slow breathing pattern associated with a “switch-off” of RSA (Rassler et al., 2018) has also been observed in a minority of fMRI subjects. Therefore, it could be speculated that low levels of anxiety do not require additional HRV increase and thus no pacemaker activity. From this point of view, we may speculate that a portion of 50% of subjects hint toward a neural pacemaker operating as a kind of “emergency system” in threat-evoking situations, when the organism is in need of additional resources.

## Limitatons and Future Prospect

This study has several limitations. A methodological limitation refers to artifacts in the ECG recording during scanning. When the scanning rate is high (more than one scan per second), artifacts might compromise the ECG recording (Kugel et al., 2003). Consequently, the detection of beat-to-beat-intervals is limited and hence, the calculation of phase-locking between BOLD and RRI signals becomes questionable. Therefore, BP beat-to-beat interval signals are also used instead of cardiac beat-to-beat intervals (Shokri-Kojori et al., 2018). However, it might be questioned whether BP and HR time series are equivalent. Note, the temporal resolution of the BOLD signal is limited to about 1 s (Buxton, 2013).

Another limitation of this protocol is that we did not measure end-tidal partial pressure of CO_2_ and can’t exclude changes of vascular responses caused by episodes of hyperventilation.

In contrast to respiration, BP and BOLD signals, which represent real analog changes in the time domain, the cardiac interval time course (RRI signal) represents beat-to-beat intervals in seconds in relation to heart beats. The two extreme cases are that the actual RR interval is dedicated either to the start of the interval or to the end of the interval. The former case is known as instantaneous RRI signal and the latter case as delayed RRI signal (De Boer et al., 1985). Therefore, a time delay of about 1 s exists between both measurements. This means, in the case of a mean pTD = 2.2 s between RRI and BOLD signals, the actual time can be longer by fractions of a second.

The Mayer waves are composed of two components originating in the baroreflex loop and in an independent central oscillator, respectively (Julien, 2006). Both components belong to the low frequency band (0.04–0.15 Hz), but there is evidence that components below and above 0.10 Hz behave clearly differently (Schwerdtfeger et al., 2020). This was an important reason for studying the band 0.10–0.15 Hz. This particular frequency band is often disregarded due to the recommendation that BOLD recordings should preferably be analyzed after low-pass filtering at 0.10 or 0.09 Hz. This low-pass filtering suppresses all frequency components above 0.10 Hz and thus, reduces artifacts (Snyder and Raichle, 2012), but also precludes the assessment of rhythmic brain stem activity.

Our findings suggest that a central pacemaker is localized in pontine or related structures. Whether such a pacemaker activates cortical structures via the ARAS (Moruzzi, 1958) remains unclear. In order to investigate such a possibility, the Granger causality principle could be used to determine causal coupling between different BOLD and RRI oscillations (Blinowska et al., 2004). The observation of time delays of similar size between RRI oscillations and distant slow BOLD signals in precentral gyrus and pons/brain stem (see Table 2) points toward a similar neural source but this has to be studied in more detail.

Remarkably, in some subjects with increased state anxiety, inspiration was associated with a HR deceleration indicating a “switch-off” of RSA (nonRSA; Rassler et al., 2018). Whether this cessation of RSA is related to the activity of a central pacemaker or to the intrinsic activity of sinoatrial node cells (Moen et al., 2019) deserves further research.

## Conclusion

(1) It could be shown that not only oscillations originating in the baroreflex loop contribute to the Mayer waves, but also central pacemaker oscillations. Such a pacemaker localized very likely in the pons/brain stem drives RRI oscillations and is associated with delayed BOLD oscillations due to the NBC time.
(2) RRI intervals are divided in LF (0.04 – 0.15 Hz) and HF (0.15 – 0.4 Hz) bands (e.g., Schwerdtfeger et al., 2020) with a boundary at 0.15 Hz. Note, that just this frequency, which characterizes the “0.15 Hz rhythm” of Perlitz et al. (2004) in brain stem was frequently observed in RRI and breathing signals (Rassler et al., 2018). Intrinsic frequency clusters, centered at 0.08 Hz and 0.15 Hz in BOLD signals, were reported by Yuen et al. (2019). Important is to note that physiological rhythms (and their pacemakers) operate over frequencies that are not controlled in rigid manner. While oscillations of BP and RRI operating in the LF band with origin in the baroreflex loop (Julien, 2006) are well established, the “0.15 Hz rhythm” in brain stem needs special attention. In our fMRI subjects, we observed two main slow frequencies in the RRI and BOLD oscillations, one with an average frequency of about 0.10 Hz, the other one with a frequency around 0.15 Hz. These oscillations represent the activity of the hypothesized pacemaker in the brain stem. In contrast, oscillations in the frequency band below 0.10 Hz were almost completely absent. Therefore, we defined the limits of our frequency band of interest by the averages of the two dominant slow rhythms. However, we plan to reassess the frequency limit at 0.15 Hz in the near future. Moreover, these two main slow frequencies document that the recommended lowpass filtering of BOLD signals at 0.10 Hz (Snyder and Raichle, 2012) may lead to a loss of information about slow oscillations that are of neural origin.
(3) The time delay between RRI and BOLD oscillations at ∼ 0.10 Hz with pTD = 2.2 ± 0.2 s found in 12 out of 23 subjects is particularly interesting. This finding strongly supports not only the existence of a central neural oscillator, but also documents that the neurovascular coupling time of 2–3 s in humans matches the delay between gamma power and BOLD signals of ^∼^2.6 s in awake mice (Mateo et al., 2017). Thus, our study provides the first evidence that the hemodynamic delay between intrinsic neural and BOLD oscillations is of similar size in different species and ranges between 2 and 3 s in awake humans.
(4) The results suggest that a method is available to assess sources of neural oscillations in the pons/brain stem

(central pacemaker) indirectly by analyzing phase coupling between cardiac beat-to-beat-intervals recorded from thorax and BOLD signals as a surrogate indicator.

## Data Availability Statement

All datasets presented in this study are included in the article/supplementary material.

## Ethics Statement

The studies involving human participants were reviewed and approved by Ethics Committee at the University of Graz. The patients/participants provided their written informed consent to participate in this study.

## Author Contributions

GP conceptualized and drafted the original manuscript. BR and AA contributed to the methodology, data processing, statistics, writing, and visualization. AS, BR, GS, and WK reviewed and edited the manuscript. All authors contributed to the article and approved the submitted version.

## Conflict of Interest

The authors declare that the research was conducted in the absence of any commercial or financial relationships that could be construed as a potential conflict of interest.

## Abbreviations

(f)MRI: (functional) magnetic resonance imaging
AAL: automated anatomy labeling
ARAS: ascending reticular activation system
AS: state anxiety
BOLD: blood-oxygenation-level-dependent
BP: blood pressure
ECG: electrocardiogram
EcoG: electrocorticogram
EEG: electroencephalogram
HR: heart rate
HRV: heart rate variability
NBC: neuro-BOLD coupling
nTD: negative time delay
PLV: phase-locking value
pTD: positive time delay
ROI: region of interest
RRI: beat-to-beat interval
RSA: respiratory sinus arrhythmia
SSMTD: subject-specific maximal pTD.
